# Suppression of TGFβ-mediated conversion of endothelial cells and fibroblasts into cancer associated (myo)fibroblasts via HDAC inhibition

**DOI:** 10.1038/s41416-018-0072-3

**Published:** 2018-04-26

**Authors:** Dae Joong Kim, James M. Dunleavey, Lin Xiao, David W. Ollila, Melissa A. Troester, Carol A. Otey, Wei Li, Thomas H. Barker, Andrew C. Dudley

**Affiliations:** 1 Department of Microbiology, Immunology, and Cancer Biology, The University of Virginia, Charlottesville, VA 22908 USA; 2 National Cancer Institute, Tumor Angiogenesis Unit, Center for Cancer Research, Frederick, MD 21702 USA; 3 Children’s Cancer Institute, Kensington, NSW 2750 Australia; 4 Department of Surgery, The University of North Carolina at Chapel Hill, Chapel Hill, NC 27599 USA; 5 Gillings School of Global Public Health, The University of North Carolina at Chapel Hill, Chapel Hill, NC 27599 USA; 6 Department of Cell Biology and Physiology, The University of North Carolina at Chapel Hill, Chapel Hill, NC 27599 USA; 7 Department of Biomedical Engineering, The University of Virginia, Charlottesville, VA 22908 USA; 8 Emily Couric Cancer Center, The University of Virginia, Charlottesville, VA 22908 USA

**Keywords:** Cancer microenvironment, Haematopoiesis

## Abstract

**Background:**

Cancer-associated fibroblasts (CAFs) support tumour progression and invasion, and they secrete abundant extracellular matrix (ECM) that may shield tumour cells from immune checkpoint or kinase inhibitors. Targeting CAFs using drugs that revert their differentiation, or inhibit their tumour-supportive functions, has been considered as an anti-cancer strategy.

**Methods:**

We have used human and murine cell culture models, atomic force microscopy (AFM), microarray analyses, CAF/tumour cell spheroid co-cultures and transgenic fibroblast reporter mice to study how targeting HDACs using small molecule inhibitors or siRNAs re-directs CAF differentiation and function in vitro and in vivo.

**Results:**

From a small molecule screen, we identified Scriptaid, a selective inhibitor of HDACs 1/3/8, as a repressor of TGFβ-mediated CAF differentiation. Scriptaid inhibits ECM secretion, reduces cellular contraction and stiffness, and impairs collective cell invasion in CAF/tumour cell spheroid co-cultures. Scriptaid also reduces CAF abundance and delays tumour growth in vivo.

**Conclusions:**

Scriptaid is a well-tolerated and effective HDACi that reverses many of the functional and phenotypic properties of CAFs. Impeding or reversing CAF activation/function by altering the cellular epigenetic regulatory machinery could control tumour growth and invasion, and be beneficial in combination with additional therapies that target cancer cells or immune cells directly.

## Introduction

Solid tumours are heterogeneous communities of cancer cells and cancer-supportive stromal cells; especially cancer-associated fibroblasts (CAFs).^1^ CAFs are identified by expression of alpha smooth muscle actin (SMA) and other contractile proteins and they secrete extracellular matrix (ECM) proteins, including periostin, fibronectin and collagen 1 (col1). Although subpopulations of CAFs may restrain tumour growth in certain contexts, CAFs and the “fortress” of ECM they produce adversely impact drug penetration within tumours, alters the immune landscape within the tumour microenvironment (TME), and prohibits the activity of targeted kinase inhibitors and immunotherapies.^2–5^ In addition to underlying genetic factors including mutational load, variability in CAF recruitment or differential activation of CAFs from patient-to-patient may impair the success of immune checkpoint inhibitors.^6^

Compared to their normal counterparts, CAFs are typically more contractile, they over-express pro-angiogenic, pro-inflammatory, and immunosuppressive cytokines, and they deposit abundant ECM that contributes to desmoplasia and fibrosis. Thickened sheets of ECM compress intra-tumoural vasculature diminishing blood flow and impairing drug delivery, while cross-linked and “stiffened” ECM creates an aberrant signalling scaffold for cancer cells and other stromal cells that fuel tumour growth.^7–9^ CAFs also appear early during tumour progression, they have a multi-source origin, including bone marrow and diverse tissue resident cell types, and they are educated by cancer cells to produce tumour-supportive factors in the tumour microenvironment (TME).^10–12^ In vitro cultured fibroblasts or endothelial cells (ECs) differentiate into CAF-like cells in the presence of inflammatory cytokines, hypoxia, biomechanical forces, and members of the TGFβ superfamily.^13^ The conversion of non-CAFs into CAFs occurs through a coordinated action of transcriptional activators/repressors in addition to genome-wide epigenetic reprogramming mediated by miRNAs and DNA/histone modifying enzymes, especially histone deacetylases (HDACs).^14^

HDACs typically repress gene transcription by deacetylating-specific lysine residues on core histone substrates; whereas, histone acetyltransferases (HATs) add acetyl groups to specific lysines thereby enabling transcriptional activation. It has recently been recognised that the epigenetic regulation of gene expression in this way, or through altered DNA methylation, imparts reversible transitions between different cellular states but may also produce stable changes in phenotype that are transmittable to cellular progeny.^15–17^ A good example is the persistent expression of genes associated with epithelial-to-mesenchymal transition (EMT) in tumours even when the initiating signals are no longer present.^18,19^ Increased expression of HDACs have also been observed in various cancers; thus, HDAC inhibitors (and other epigenetic modifying drugs) are currently under investigation for the treatment of both solid and haematological malignancies.^20^ Most of these reagents are designed to target-specific epigenetic modifications in cancer cells that contribute to their growth and survival; however, few studies have focused on auxiliary cell types in the TME, for example CAFs, as indirect targets of their pharmacological activity.

Here we have used freshly isolated ECs and bona fide CAFs to explore the epigenetic pathways that promote non-CAF to CAF conversion or maintain the phenotypic and functional properties of CAFs. We have identified Scriptaid (a selective inhibitor of HDACs 1, 3, and 8) as a potent reagent that reverses several well-known CAF features including their enhanced contractility, abundant ECM expression, and TGFβ pathway activation. Scriptaid also impairs CAF’s tumour-supportive properties in vitro and in vivo; thus, Scriptaid or similar HDAC inhibitors may represent a class of molecular therapeutics that target both cancer cells and stromal cells in the microenvironment of solid tumours.

## Materials and methods

### Antibodies and materials

Recombinant TGFβ2 was purchased from PeproTech (Rocky Hill, NJ). Scriptaid, MS-275, PCI34051, and Pyroxamide were purchased from Tocris (Ellisville, MO). CUDC907 was obtained from Selectchem (Houston, TX). Nexturastat A was from Biovision Inc (Milpitas, CA). RGFP966 was purchased from MedKoo Bioscience (Morrisville, NC). Other HDAC inhibitors were provided free of charge by the UNC Drug Discovery Core at UNC Chapel Hill. Monoclonal SMA antibody was purchased from Sigma-Aldrich (St Louis, MO). GAPDH antibody was obtained from Cell Signaling (Beverly, MA). The rabbit polyclonal anti-H3K4, 9, and 27 antibodies were from Active Motif (Carlsbad, CA). Fibronectin and collagen type I antibodies were from Abcam (Cambridge, MA). Palladin antibodies, pan-palladin, and palladin isoform 3 and 4 antibodies were generated by Dr. Carol A. Otey.

### Cell lines, cell culture, and media

Endothelial cells were isolated and cultured in a defined culture medium as previously described.^13,21,22^ Briefly, C3-TAg tumours were resected, minced into ~3 mm pieces, and digested using an enzymatic cocktail followed by mechanical disruption. Homogenised samples were filtered (using a 100 μM strainer) and then resuspended in MACS buffer (degassed PBS containing 2 mM EDTA and 0.5% BSA), and endothelial cells were captured using immunomagnetic selection with CD31 antibodies. After washing and eluting the CD31^+^ fractions, endothelial cell colonies were selected based on positive uptake of DiI-Ac-LDL. Colonies were further expanded and then characterised by qPCR for expression of CD31 and VE-cadherin and absence of CD45 expression. Colonies were also routinely characterised for CD31 expression by immunofluorescence and flow cytometry as we previously described.^21^ To induce CAF-like differentiation, the medium was removed and replaced with 20% FBS in DMEM. The cells were then incubated for 48 h in the presence of 10 ng/mL TGFβ mCAFs and hCAFs were cultured DMEM with 20% FBS plus bFGF. mCAFs were previously isolated by us or were isolated from BRAF^mut^ mice by digesting tumours in a collagenase/dispase/DNAse cocktail for 30 min with shaking at 37 °C, filtering through a 100 μM filter, and seeding cells in DMEM with 10% FBS and 10 ng/mL bFGF.^23^ CAFs were characterised by immunocytochemistry, western blotting, and by qPCR using at least three markers, including *Col1*, *SMA*, and *Fn*. hCAFs were isolated and described previously.^24^ All tumour cell lines were cultured with 10% FBS in DMEM.

### Immunoblotting

Preparation of cell extracts and western blotting was carried out as previously described by us using standard methodologies.^13^

### Real-time quantitative PCR (qPCR)

RNA extraction and qPCR were carried out as previously described by us.^13^

### Microarray and analysis

RNA was collected from mCAFs treated with Scriptaid or DMSO vehicle control for 7 days and run on a GeneChip Mouse Transcriptome Array 1.0 Chip (Affymetrix). The data were analysed using Expression Console software (v. 1.4.1) for quality control and Transcriptome Analysis Console software (v. 3.0.0.466) for gene expression level changes. Hierarchical clustering analysis and pathway analysis were carried out using WikiPathways (www.wikipathways.org).

### Collagen gel contraction assay

The collagen gel assay was performed following the manufacturer’s instructions (Cell Biolabs). Briefly, 24-well culture plates were pre-coated with 5% BSA in PBS. Collagen gels were polymerised for 1 h at 37 °C. Cells were seeded on the collagen gel and incubated for 4 h. After 4 h of incubation, 1 ml of DMEM (20% FBS) media containing 10 ng/mL TGFβ, Scriptaid, or Scriptaid plus TGFβ were added to each well and incubated at 37 °C for 48 h. For mCAFs, CAFs were mixed with collagen gel and then polymerised for 1 h at 37 °C. Cells were incubated for 48 h in the presence of vehicle or Scriptaid. Gels were released from the wells and the percentage of contraction was measured. A minimum of three collagen gels was assayed per experimental condition. The area of the collagen gel contraction was determined by Metamorph software and the relative changes in the surface area were plotted.

### Atomic force microscopy

Atomic force microscopy was carried out as described previously.^25^ Briefly, Scriptaid treated or vehicle-treated mCAFs were subjected to a MFP-3D Atomic Force Microscope (AFM) (Asylum Research) with a TiE inverted optical microscope (PlanFluor 40, 0.6 NA objective) (Nikon). A silicon nitride AFM cantilever (MLCT-O10, Bruker, Billerica, MA) glued with a 6 μm diameter polystyrene sphere was used. The spring constant of the cantilever was in the range of 0.01–0.02 N/m determined by the thermal resonance frequency method. A Hertz model of contact mechanics of a sphere and an elastic material was used to analyse Force-Indentation curves with a Poisson ratio of 0.5.

### 2D CAF/tumour cell spheroid assay

pLV Azurite and pLV Cherry constructs were purchased from Addgene. psPAX2, pMD2.G, and the vector constructs were transfected into 293T cells using a standard calcium phosphate method. Lentivirus particles were collected from the filtered conditioned medium using the Lenti-X-concentrator (Clonetech). To generate fluorescence expressing cell lines, tumour cells or CAFs were infected with lentivirus in DMEM plus polybrene.

For 2D tumour spheroid co-culture assays, D4M^Azurite^ and mCAFs^mCherry^ were cultured to 70% confluence. D4M^Azurite^ cells (1.5 × 10^5^ cells/ml) were seeded in 20 µl hanging drops containing 5% methylcellulose on the inverted lid of the 10 mm petri dish containing PBS. The dishes were incubated for 24 h to allow aggregation into the spheroid. Generation of tumour spheroids was confirmed under light microscopy. mCAFs^mCherry^ were seeded at a 1.5 × 10^5^ cells/ml in six-well plates. Scriptaid treated (7 days) mCAFs^mCherry^ were extensively washed to remove any residual Scriptaid. Tumour spheroids were then collected and transferred to six-well plates previously seeded with control or Scriptaid-treated mCAFs^mCherry^. The cultures were placed at 37 °C in a humidified incubator under a 5% CO_2_ atmosphere for 48 h. Images were analysed using NIS Elements software (Nikon).

### ECM deposition assay

The ECM deposition assay was performed as described previously.^26^ Briefly, mCAFs were seeded (1.5 × 10^5^) and incubated for 7 days (control versus Scriptaid or with the indicated si-RNAs) to allow for secretion and deposition of ECM. Dishes were treated with ammonium hydroxide based extraction buffer for 5 min and then rinsed in PBS. In some experiments, tumour spheroids were seeded on top of the intact ECM and allowed to adhere. The length and area of tumour cell invasion were determined as described above. For immunofluorescence, the deposited ECM was fixed with 2% PFA and incubated in blocking buffer for 30 min to reduce non-specific antibody binding. Incubation with the primary antibody was carried out for 1 h, followed by secondary antibodies for 50 min at room temperature. Stained samples were mounted in Vectashield (Vector Laboratories, Burlingame, CA, USA).

For cell length analysis, D4M melanoma cells (1 × 10^4^) were plated on the deposited ECM produced by control or Scriptaid treated mCAFs in Dulbecco's modified Eagle's medium (DMEM w/o phenol red) supplemented with 10% foetal calf serum and penicillin (100 U/ml)/streptomycin (100 mg/ml) for 48 h at 37 °C, 5% CO_2_ and 70% humidity. Cells were imaged every 10 min in bright field at ×10 magnification for 24 h using the Operetta imaging system (PerkinElmer). For image analysis, captured images were analysed by NIS element software (Nikon).

### Immunohistology

Freshly resected tumours were fixed overnight in 4% PFA/PBS. The next day, tumours were moved to a 30% sucrose solution for an additional 24 h before embedding in OCT. Cryosections were mounted in Vectashield containing DAPI and immediately viewed and photographed using a Nikon Eclipse Ti-E inverted microscope and NIS-Elements software package. For immunocytochemistry, cells were seeded in chamber slides (EMD Millipore) for 48 h, washed with PBS, and fixed with 4% formaldehyde for 10 min at room temperature before blocking and staining overnight with primary antibodies. After secondary antibody labelling and washing, slides were mounted and images were captured as described above.

### Tumour studies in mice

All experiments were performed in accordance with the University of North Carolina at Chapel Hill and the University of Virginia guidelines for animal handling and care. One million murine B16F10 tumour cells were injected subcutaneously into the skin of 8-week-old SMA-DsRed:Col1-GFP mice (these mice were provided by Dr. Scott Magness and Dr. Kay Lund and were described previously).^27,28^ Mice were treated i.p. with Scriptaid, dissolved in DMSO, 3× per week (5.5 mg/kg). Human A375SM melanoma cells were injected subcutaneously into nu/nu mice alongside one million partnering mCAFs that were previously isolated by us from the same tumour model.^23^ Individual tumour sizes were measured with calipers each day. At the end of the experiment, mice were killed and tumours were collected and weighed. Collagenase-dispersed tumours were analysed by flow cytometry as previously described by us except no antibody staining was required due to the native fluorescence of the GFP/DsRed reporter genes.^29^

## Results

### A drug screen identifies Scriptaid as an inhibitor of de novo CAF characteristics in non-CAFs

**Fig. 1:**
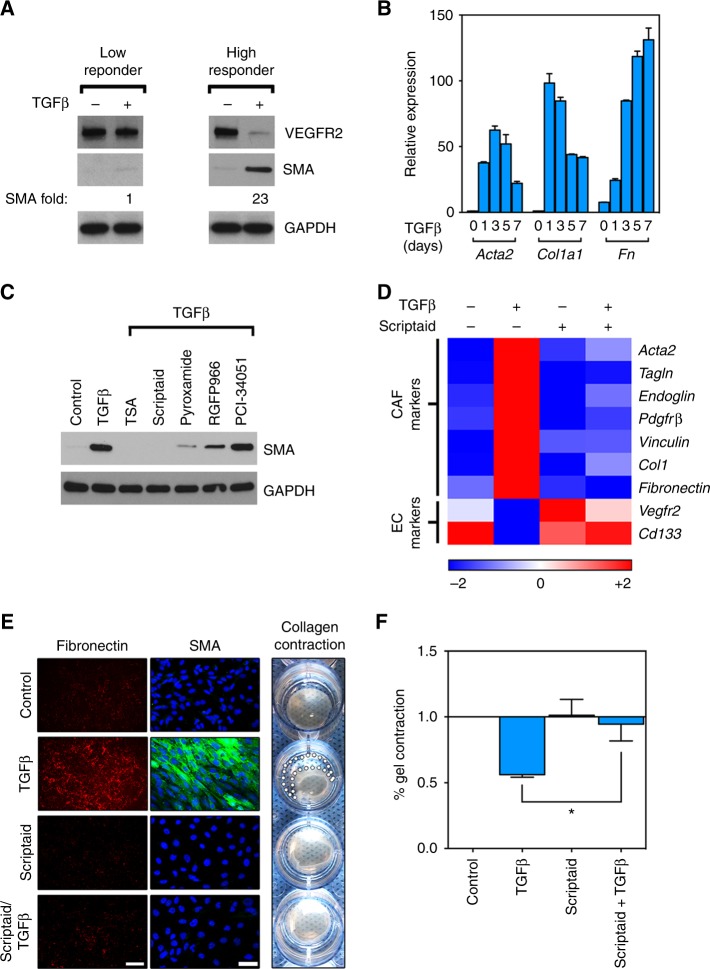
A drug screen identifies Scriptaid as an inhibitor of de novo CAF characteristics in non-CAFs. **a** A TGFβ low responder or high responder challenged with 10 ng/mL TGFβ for 48 h. Cell lysates were analysed by western blot. **b** A TGFβ high responder was treated with TGFβ for the indicated time points, total RNA was collected and reverse transcribed, and subjected to qPCR (results are means ± SEM). **c** TGFβ high responder treated with TGFβ in addition to 2 μM TSA (pan HDAC inhibitor), 2 μM Scriptaid (HDAC 1/3/8 inhibitor), 1.5 μM Pyroxamide (HDAC 1 inhibitor), 1.5 μM RGFP966 (HDAC 3 inhibitor), or 5 μM PCI34051 (HDAC 8 inhibitor) for 48 h. Cell lysates were analysed by western blot. **d** TGFβ high responder treated with TGFβ, Scriptaid, or the combination. Relative mRNA expression was measured by qPCR and is presented as a heat map (log2). **e**, **f** ECM deposition assay was performed and plates were stained with fibronectin antibody (left panels). In separate plates, cells were fixed and stained with SMA antibody (middle panel), or analysed in a collagen gel contraction assay which is quantified at far right, *P < 0.05 when comparing Scriptaid-treated versus TGFβ and Scriptaid treated wells by Student’s *t*-test. Methodology for quantification of gel contraction assays is described in the Methods section. Scale bar is 100 μm

We recently reported that subpopulations of tumour-derived endothelial cells (TECs) are heterogeneous in their ability to differentiate into SMA^+^ CAF-like cells after TGFβ stimulation.^13,21,22^ Because normal fibroblasts will spontaneously gain CAF markers upon in vitro culture, we reasoned that some of these TEC precursors, being absent for SMA and other CAF markers even after in vitro propagation, would be a useful tool for studying how non-CAFs are converted to CAFs by TGFβ. At least two subpopulations of TECs have been identified and we have termed these TGFβ “low responders” versus “high responders.” In contrast to TGFβ low responders, TGFβ high responders show rapid and robust (23-fold) upregulation of SMA protein expression by western blot (Fig. 1a). Using qPCR, we also observed that mRNAs for CAF markers (*Acta2*, *Col1*, *Fn*) were strikingly upregulated after TGFβ challenge suggesting that de novo CAF markers could be readily induced in “high responder” TECs (Fig. 1b).

Next, we screened a panel of compounds for activity against TGFβ-induced conversion of non-CAFs into SMA^+^ CAFs (summarised in Supplementary Table 1). From our screen, we turned our attention to Scriptaid because it was the most effective HDAC inhibitor identified in our study, and it was previously shown to be well-tolerated in mice.^30^ Scriptaid was equally effective as the pan-HDAC inhibitor TSA in blocking TGFβ-induced SMA expression, whereas HDAC 1 (Pyroxamide) and HDAC 3 (RGFP966) inhibitors were moderately effective. An HDAC 8 inhibitor was ineffective at blocking SMA expression (Fig. 1c). A panel of TGFβ-induced CAF marker genes was also blocked by Scriptaid including mRNAs for *Acta2*, *Tagln*, *Endoglin*, *Pdgfrβ*, *Vinculin*, and *Col1*. On the other hand, the expression of EC markers that are typically downregulated by TGFβ were maintained suggesting that inhibiting specific HDACs blocks the TGFβ-driven conversion of non-CAFs into CAFs (Fig. 1d). Scriptaid also reduced fibronectin secretion, diminished SMA^+^ stress fibres measured by immunofluorescence, and reversed TGFβ-induced collagen gel contraction which are all cardinal CAF features (Fig. 1e, f).^31^ Taken together, Scriptaid potently inhibits de novo expression of genes that typify and functionalise SMA^+^ CAFs.

### Scriptaid reverses the expression of SMA and ECM components and reduces CAF contractility and stiffness

**Fig. 2:**
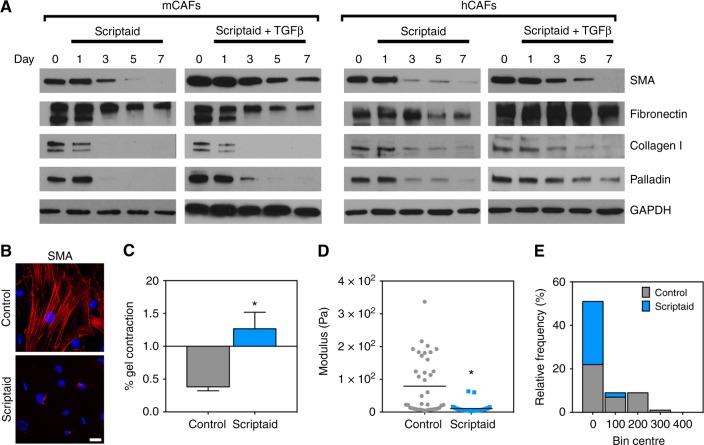
Scriptaid reverses the expression of SMA and ECM components and reduces CAF contractility and stiffness. **a** Murine melanoma CAFs (mCAFs) and human breast CAFs (hCAFs) were treated with or without 10 μM Scriptaid and TGFβ for 4 days and then sub cultured for an additional 3 days. CAF marker expression was measured by western blot. **b** SMA^+^ stress fibres in control mCAFs or mCAFs treated with Scriptaid. **c** Gel contraction assay in mCAFs quantified as described in the Methods section, *P < 0.05. **d**, **e** Modulus of mCAFs treated with Scriptaid was measured by AFM (MFP-3D) with a MLCT-O10 cantilever (spring constant: 0.01–0.02 N/m) glued with a 6 µm polystyrene bead. The modulus of single CAFs was analysed by a Hertz model with a Poisson ratio of 0.5. “Stiffening” is reported in Pascals (Pa) and is relative to vehicle-treated CAFs, *P < 0.01

We next tested whether Scriptaid could revert freshly isolated CAFs that constitutively express CAF marker genes. We treated murine melanoma CAFs (mCAFs) or human breast CAFs (hCAFS) with Scriptaid over a 7-day period in the presence or absence of TGFβ (these CAFs were previously isolated and characterised by our laboratories).^23,24^ We observed that Scriptaid reduced the expression of SMA, fibronectin, col1, and the cytoskeletal protein palladin^32^ both in the presence and absence of TGFβ (Fig. 2a). Identical results were obtained using murine mammary CAFs and human melanoma CAFs (data not shown). Scriptaid also completely abolished contractile SMA^+^ stress fibres determined using immunofluorescence microscopy (Fig. 2b). The loss of incorporation of SMA into stress fibres accompanied a reversal of CAF-mediated collagen gel contraction and an ~50% reduction in cellular stiffness as determined using atomic force microscopy (Fig. 2c–e). Thus, Scriptaid reverses some of the cardinal features of bona fide CAFs, including their abundant ECM expression, organisation of SMA into highly contractile stress fibres, and biomechanical stiffness.

### CAFs independently treated with Scriptaid impair collective cell invasion in CAF/tumour cell spheroid co-cultures

**Fig. 3:**
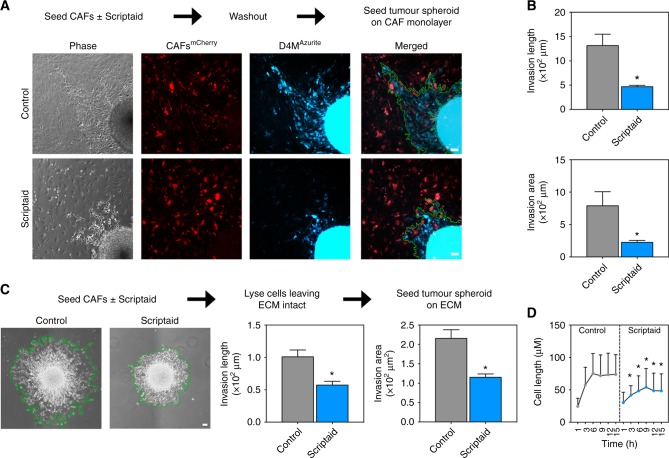
CAFs independently treated with Scriptaid impair collective cell invasion in CAF/tumour cell spheroid co-cultures. **a** Experimental strategy and spheroid images. mCAFs^mCherry^ were treated with 10 μM Scriptaid for 7 days and then washed exhaustively. Spheroids of D4M^Azuirte^ tumour cells prepared separately were then seeded on CAF monolayers. **b** Quantification of results (Methods section). Results were quantified from three independent experiment and analysed by Student’s *t*-test. Data are mean ± SEM, *P < 0.05. **c** Spheroids of D4M tumour cells prepared separately were seeded on intact ECM isolated from mCAF monolayers (control versus Scriptaid-treated). Results (invasion length and area) were quantified from three independent experiments. Data are mean ± SEM, *P < 0.05. **d** Time-lapse images from tumour cells seeded on ECM from control versus Scriptaid-treated CAFs. Between 80 and 200 cells were analysed for each time point and results compared using Student’s *t*-test. Data are mean ± STD, *P < 0.05

To determine whether Scriptaid-treated CAFs lose their tumour-supportive properties including the ability to promote collective tumour cell invasion,^33^ we generated two different co-culture systems. Here we used freshly isolated mCAFs from murine melanoma using BRAF^mut^ mice alongside D4M melanoma cells derived from the same tumour model.^34^ First, after labelling cells with fluorophores using lentivirus, we treated mCAFs^mCherry^ for 7 days with Scriptaid followed by extensive washing to remove any residual drug. Next, D4M^Azurite^ spheroids were seeded on CAF^mCherry^ monolayers and left untreated for 24 h. Using two different metrics including invasion length and area, the results showed a 2–3-fold reduction in D4M tumour cell invasion when spheroids were plated on Scriptaid-treated versus control CAF monolayers (Fig. 3a, b). Second, we tested whether isolated ECM from control versus Scriptaid-treated mCAFs would differentially affect tumour cell invasion. To enrich deposited ECM, mCAFs were incubated for 7 days in the presence or absence of Scriptaid and then removed by ammonium hydroxide extraction buffer leaving the intact ECM adhered to the culture dish. The results showed an approximately twofold reduction in tumour spheroid invasion on ECM deposited by Scriptaid-treated versus vehicle-treated mCAFs (Fig. 3c). Time-lapse images taken over a 15 h period confirmed that D4M cancer cells were approximately twice as large, indicating enhanced spreading and attachment, when seeded on ECM from control versus Scriptaid-treated CAFs (Fig. 3d). Taken together, these results suggest that Scriptaid impairs the ability of CAFs to secondarily support collective tumour cell invasion in vitro that is independent of a direct effect of Scriptaid on cancer cell survival or motility.

### A genome-wide array shows inhibition of the TGFβ pathway, ECM components, and contractility in Scriptaid-treated mCAFs

**Fig. 4:**
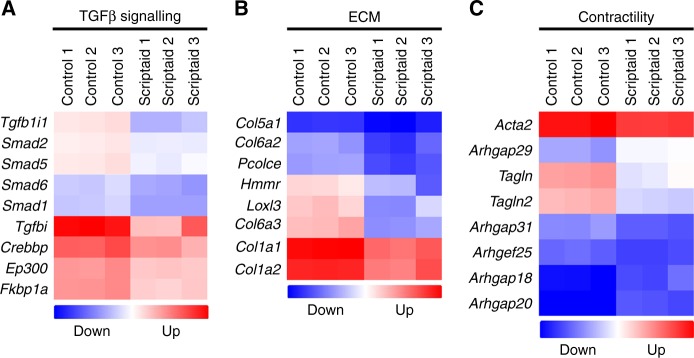
A genome-wide array shows inhibition of the TGFβ pathway, ECM components, and contractility in Scriptaid-treated mCAFs. mCAFs were treated with Scriptaid (in triplicate) and total RNA was analysed using Affymetrix Mouse Gene 1.0 ST microarrays. With a threshold set at twofold differences between control versus Scriptaid-treated mCAFs, genes were clustered and separated into those involved in **a** TGFβ signalling **b** ECM production, and **c** contractility. Results were log2 transformed and upregulated genes are indicated in red; downregulated genes in blue

Next, mCAFs challenged with Scriptaid were subjected to an Affymetrix Mouse Transcriptome Array 1.0 to examine global changes in gene expression. As expected, perturbation of histone acetylation with Scriptaid resulted in changes in the expression of hundreds of genes globally; in particular, there was repression of several selected genes related to TGFβ signalling (e.g., *Smad* transcription factors and the histone acetyltransferase *Ep300*), ECM (e.g., *Col1a1* and *Col1a2*), and cellular contractility (e.g., *Acta2*, *Tagln*/*Tagln2*, and several guanine exchange factors) (Fig. 4a–c). In addition to inhibition of factors important for cytoskeletal organisation, the microarray also showed both inhibition and stimulation of several known cytokines and chemokines, many of which have roles in the mobilisation and activation of different immune cell populations including T^reg^ and T^effector^ cells or are important components of the SASP (senescence-associated secretory phenotype) (Figure S1). Thus, Scriptaid targets multiple CAF effector pathways simultaneously showing selectivity for TGFβ signalling, ECM production, and cellular contraction all of which are important for CAF functions in solid tumours.

### Scriptaid delays tumour growth and diminishes CAF abundance in a murine tumour model

**Fig. 5:**
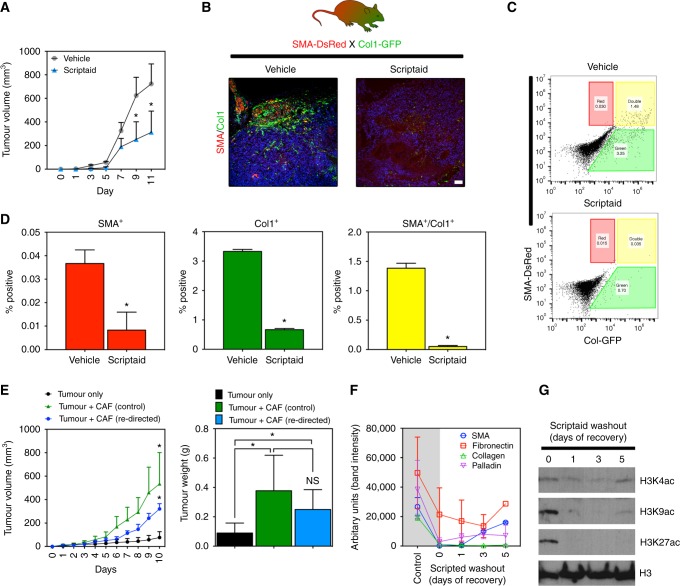
Scriptaid delays tumour growth and diminishes CAF abundance in a murine tumour model. **a** Scriptaid delays the growth of B16F10 melanoma. Mice were treated i.p. with 5.5 mg/kg Scriptaid or vehicle (PBS/DMSO) 3×/week. Tumour sizes were determined using calipers, *n* = 5 mice per group. Data are mean ± SEM, *P < 0.05 where indicated analysed by Student’s *t*-test. **b** Immunohistology in tumours from vehicle-treated versus Scriptaid-treated SMA-DsRed:Col1-GFP reporter mice. Nuclei were counterstained with DAPI. **c** FACS analysis in collagenase-dispersed tumours from reporter mice. **d** Quantification of FACS analysis (*n* = 3 individual tumours per group). Data are mean ± SEM, *P < 0.05 where indicated as analysed by Student’s *t*-test. **e** Tumour cells only, tumour cells partnered with control mCAFs, or tumour cells partnered with mCAFs treated with Scriptaid in vitro for three weeks were co-injected subcutaneously into nu/nu mice and tumour volumes and weights were recorded (*n* = 5 mice per group). Data are mean ± SEM, *P < 0.05 (*n* = 5). **f** mCAFs treated with Scriptaid for three weeks were washed and re-seeded into their normal growth medium without Scriptaid. Cell lysates were prepared on the indicated day and then analysed for protein expression by western blotting. Results are presented as the average of two independent experiments (band density was measured using ImageJ analysis software). **g** Histone acetylation determined by western blotting on the same samples shown in **f**

We tested the effect of Scriptaid on murine melanoma (B16F10) growth and CAF abundance in vivo using reporter mice that have fibroblasts genetically marked (SMA-DsRed:Col1-GFP mice).^27,28^ In this model, i.p. administration of Scriptaid inhibited tumour growth by ~twofold at the end of the experiment and diminished SMA^+^/Col1^+^ CAFs indicated by a clear reduction in DsRed/GFP signals on fresh cryosections (Fig. 5a, b). Using FACS, we observed quantitative reductions in SMA^+^ and Col1^+^ populations; in particular, (myo)fibroblasts double positive for SMA and col1 (which typically identify CAFs) were reduced ~40-fold (Fig. 5c, d). To determine Scriptaid’s direct effect on CAF function(s) in vivo, we challenged mCAFs with Scriptaid or vehicle in vitro and then co-engrafted them with human A375 melanoma cells in immune-deficient mice. As expected, control CAFs (vehicle-treated) enhanced tumour growth compared to cancer cells injected without partnering CAFs. In contrast, Scriptaid-treated CAFs were less effective at supporting tumour growth and instead had intermediate tumour-supportive abilities (Fig. 5e). We therefore reasoned that Scriptaid-treated CAFs might revert to their activated phenotype in the absence of continuous Scriptaid treatment. Indeed, after washing out Scriptaid from CAFs that were Scriptaid-treated for a prolonged period, the expression of CAF markers, with the exception of col1, were rapidly re-gained although their level of expression did not reach base-line controls (Fig. 5f). Cryosections from these tumours confirmed that SMA expression was present in all three experimental groups indicating that CAFs rapidly regain SMA expression in vivo once Scriptaid is removed. Notably, CAF recovery occurs in vivo despite a direct anti-proliferative effect on CAFs in vitro indicating that once Scriptaid is removed our diluted out, CAFs quickly regain their proliferative ability (figure S2). Histone acetylation was also rapidly lost upon Scriptaid washout (Fig. 5g); thus, while Scriptaid may re-direct CAFs into a SMA^−^ phenotype with reduced ECM secretion, continuous Scriptaid administration may be necessary to convert CAFs into functionally dissimilar non-CAFs over prolonged periods.

### Silencing HDACs 1, 3, and HDAC 8, is sufficient to suppress the expression of several CAF marker genes

**Fig. 6:**
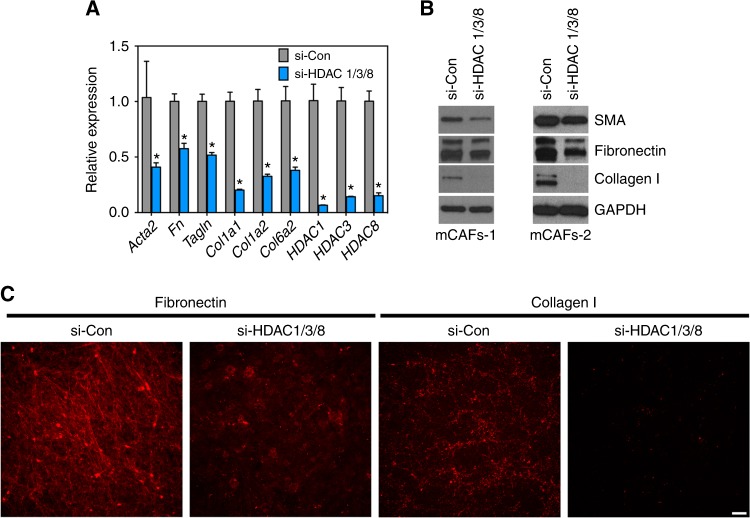
Silencing HDACs 1, 3, and HDAC 8 is sufficient to suppress the expression of several CAF marker genes. **a** mCAFs were transfected with siRNA scrambled negative control or si-HDAC 1/3/8 two times over a 7 day period. Gene expression was analysed by qPCR at the end of the experiment. Results are presented as mean ± SEM, *P < 0.05 using Student’s *t*-test. **b** Cell lysates from the same experiment were analysed by western blotting for SMA, fibronectin, and col1. **c** ECM deposition assay was carried out in cells treated as described in **a**

Finally, because Scriptaid is a pharmacological inhibitor with selectivity for HDACs 1, 3, and 8, we asked whether silencing these HDACs would impact the expression of commonly accepted CAF marker genes. To mimic the treatment strategy used for Scriptaid, mCAFs were treated with two rounds of HDACs 1, 3, and 8 siRNAs over 6 days followed by RNA purification and qPCR analysis. We focused on the CAF marker genes encoding ECM components identified from our microarray analysis shown in Fig. 4. The results show that silencing these HDACs in combination was sufficient to produce a 25–50% reduction in the expression of selected CAF markers (Fig. 6a). Western blotting of whole-cell lysates confirmed that silencing HDACs 1, 3, and 8 was also sufficient to reduce the protein expression of SMA, fibronectin, and col1 although this effect was not as robust as adding Scriptaid directly to CAF cultures (Fig. 6b). Using an ECM deposition assay whereby CAFs are removed by ammonium hydroxide lysis, secreted fibronectin and col1 adhered to tissue culture dishes was also reduced in siRNA-treated mCAFs (Fig. 6c). These results suggest that Scriptaid reverses expression of several ECM components and SMA in CAFs predominately through targeting HDACs 1, 3, and 8; however, because HDAC silencing does not entirely phenocopy the potent effect of Scriptaid, we cannot rule out the possibility that Scriptaid also acts on additional HDACs or non-histone substrates in tandem, including several important effectors of the TGFβ signalling pathway.

## Discussion

HDAC inhibitors are new and emerging cancer therapies; however, few studies have focused on the TME as an endpoint for their activity.^35^ We show that Scriptaid antagonises TGFβ-mediated CAF gene expression, which accompanies diminished ECM deposition, reduced contractility, and ECM stiffness, and delayed tumour growth in vivo; thus, Scriptaid potentially reverses a tumour-permissive microenvironment by reducing ECM accumulation preventing activation of intrinsic mechanosensory pathways in CAFs and cancer cells that promote cancer progression. However, the use of HDAC inhibitors for the treatment of solid tumours must be used with caution as recent studies have suggested that HDAC inhibition (in particular HDAC2) may also increase the expression of SASP factors that could be tumour supportive.^36,37^ We also found enhanced expression of several cytokines and chemokines in Scriptaid-treated CAFs some of which have known tumour-supportive functions, others with well-known tumour-suppressive functions, and some which have both.^38^ A better understanding of the specific HDAC-mediated pathways that functionalise tumour-supportive CAFs will therefore aid in the rational use of HDAC inhibitors with balanced selectivity for these specific pathways. For example, because CAFs and the ECM they produce are suggested to provide “safe havens” for cancer cells, judicious use of an HDAC inhibitor that selectively blocks ECM, without stimulating tumour-supportive cytokine expression, might prove beneficial when combined with immunotherapy, chemotherapy, or other targeted kinase inhibitors.^5,39^

In addition to abundant expression of SMA and ECM, the persistent activation of pathways that drive cellular contraction is a cardinal feature in CAFs, which contributes to the reported matrix “stiffening” in solid tumours.^7,40^ For example palladin isoform 3, which we show is reduced by Scriptaid, influences the contractile machinery in CAFs by incorporating into SMA^+^ stress fibres to enhance actin bundling.^41^ Consistent with this observation, introduction of palladin into human dermal fibroblasts was shown to promote their activated (myo)fibroblastic transition which supports cancer cell invasion.^42^ Upregulated palladin in CAFs also secondarily regulates expression of additional cytoskeleton-associated proteins, including the small GTPase Cdc42, to promote cancer cell invasion and metastasis.^43^ Based on our gene array analysis, Scriptaid produced a large-scale downregulation of several genes important for establishment/function of the cytoskeleton including *Acta2*, *Actg2*, *Cdc42*, *Tagln*/*Tagln2*, and several guanine exchange factors. It is likely that diminished expression of these factors by Scriptaid impairs CAF motility and contractility, which could account for the reduced collective cell invasion observed in our CAF/tumour cell spheroid co-cultures and reduced CAF “stiffness” as determined by atomic force microscopy.

While manipulation of the epigenetic programming that maintains the activation of CAFs could be a powerful strategy to impede cancer progression, a key question was how Scriptaid blocked the conversion of non-CAFs into CAFs or reversed the activation of freshly isolated CAFs. Similar to our study, other HDAC inhibitors and additional drugs that target the epigenome were shown to block (myo)fibroblast activation and/or smooth muscle cell differentiation programs.^44–48^ But it was unclear whether the same HDAC-mediated pathways that promote activation of (myo)fibroblasts would also be operative in bona fide CAFs or ECs.^45,49,50^ We show that manipulation of three HDACs either with Scriptaid or with siRNAs is sufficient to reverse many of the cardinal features that typify CAFs. However, because many HDAC inhibitors have as yet undefined non-histone substrates as their targets, we cannot rule out the possibility that Scriptaid also blocks key auxiliary pathways that drive CAF function independent of HDAC-mediated regulation of gene expression. Indeed, in light of the transient inhibitory effect Scriptaid has on the expression of certain CAF marker genes, it is likely that non-histone targets, downstream of the TGFβ pathway, are dually inhibited by Scriptaid treatment. Furthermore, because sustained treatment with Scriptaid is necessary to fully maintain CAFs in their re-directed state, pharmacological reagents such as Scriptaid, while altering chromatin structure in ways that impede transcriptional activation of CAF marker genes, may not induce permanent changes that prevent re-activation of these genes. Genetic ablation of specific HDACs or other epigenetic effectors in precursor populations that generate CAFs in murine models of cancer or in three-dimensional organotypic models in vitro could directly test their role in regulating persistent CAF activation.^51^

Finally, a limitation of this study is that, similar to other pharmacological reagents, HDAC inhibitors such as Scriptaid have the potential to impact the function of multiple cell types found within the TME in ways that may inhibit^39,52^ or even support tumour progression.^36,37^ Thus, careful consideration of the dose, scheduling, and specific HDAC to be targeted should be optimised in therapeutic strategies using HDAC inhibitors alone or in combination with other drugs. Furthermore, like other HDAC inhibitors, Scriptaid has a growth inhibitory effect on CAFs; thus, we cannot rule out the possibility that Scriptaid both represses a contractile, myogenic CAF phenotype and inhibits CAF proliferation which has the cumulative effect of reducing the actual numbers of CAFs present in the TME if administered continuously. Building on our work, more targeted therapies could be developed to control (myo)fibroblast activation and proliferation in diverse disease settings including hepatic or pulmonary fibrosis, nephropathy, cardiovascular disease, and cancer.

## Electronic supplementary material

Supplemental figure legends(DOCX 59 kb)

S1(PNG 263 kb)

S2(PNG 4154 kb)

Table S1(DOCX 496 kb)

## Availability of materials

All materials and datasets will be made available by sending an email request to the corresponding author. Raw data from the array will also be deposited here (https://www.ncbi.nlm.nih.gov/geo/).
