# Supplementary material for: Suppression of TGFβ-mediated conversion of endothelial cells and fibroblasts into cancer associated (myo)fibroblasts via HDAC inhibition
Source: Br J Cancer. 2018 Apr 26;118(10):1359–68. doi: 10.1038/s41416-018-0072-3 (PMC5959903; doi:10.1038/s41416-018-0072-3)
Supplement: Supplementary file 1 — Supplemental figure legends(DOCX 59 kb) [file 41416_2018_72_MOESM1_ESM.docx]

**Supplemental figure legends**

**Table S1. Summary of HDAC inhibitors tested in this study.**

**Figure S1. Additional gene expression heat maps from vehicle-treated and Scriptaid-treated mCAFs.** (A) Cytoskeleton-associated genes. (B) Selected cytokines and growth factors. (C) qPCR analysis of SASP factors and additional cytokines/chemokines in Scriptaid-treated CAFs, n.d. = not detected. Results are presented as mean ± SEM, *P<0.05 using Student’s t-test.

**Figure S2. Despite Scriptaid’s anti-proliferative effect, CAFs recover following Scriptaid withdrawal.** (A) Expression of SMA in tumors alone, tumors where co-partnering CAFs were injected, or tumors where co-partnering CAFs treated in vitro with Scriptaid were injected. (B) Scriptaid dose response and proliferation assay in mCAFs. (C) Cleaved caspase 3 western blot in mCAFs treated with 10 μM Scriptaid for seven days. Staurosporin was used as a positive control.
