# Supplementary figures and images for: Suppression of TGFβ-mediated conversion of endothelial cells and fibroblasts into cancer associated (myo)fibroblasts via HDAC inhibition
Source: Br J Cancer. 2018 Apr 26;118(10):1359–68. doi: 10.1038/s41416-018-0072-3 (PMC5959903; doi:10.1038/s41416-018-0072-3)

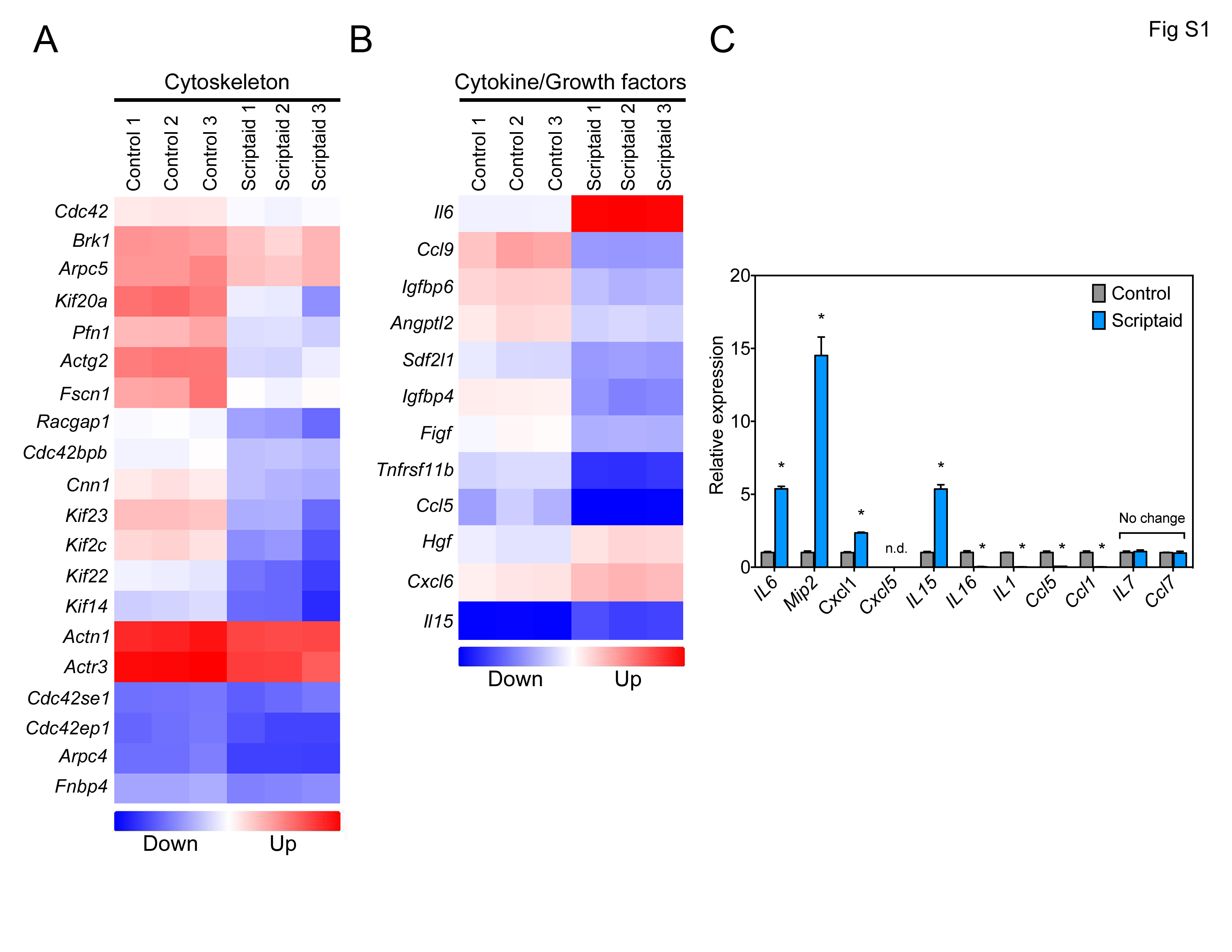

Supplement: Supplementary file 2 — S1(PNG 263 kb) [file 41416_2018_72_MOESM2_ESM.png]

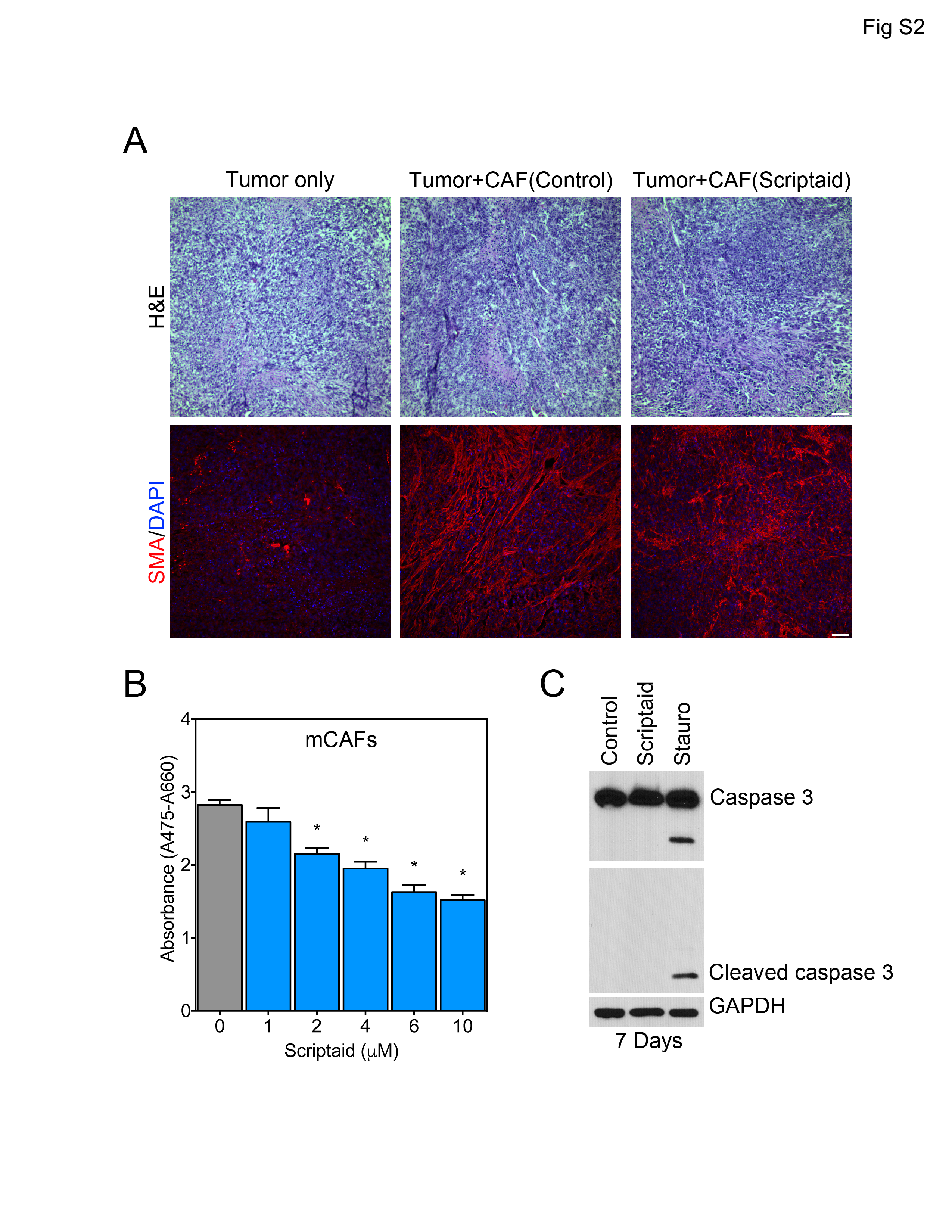

Supplement: Supplementary file 3 — S2(PNG 4154 kb) [file 41416_2018_72_MOESM3_ESM.png]
