# Supplementary material for: Suppression of TGFβ-mediated conversion of endothelial cells and fibroblasts into cancer associated (myo)fibroblasts via HDAC inhibition
Source: Br J Cancer. 2018 Apr 26;118(10):1359–68. doi: 10.1038/s41416-018-0072-3 (PMC5959903; doi:10.1038/s41416-018-0072-3)
Supplement: Supplementary file 4 — Table S1(DOCX 496 kb) [file 41416_2018_72_MOESM4_ESM.docx]

| Inhibitor | Targets | Ability to block CAF SMA expression |
| --- | --- | --- |
| MS275 | HDAC 1,3 | ++ |
| TSA | All HDACs | ++ |
| CUDC-907 | HDAC 1,3,6,10,11 | ++ |
| MC1568 | HDAC 4,5,9 | +/- |
| Droxinostat | HDAC 3,6,8 | - |
| CI994 | HDAC 1,3 | +/- |
| MGCD0103 | HDAC 1,2,3,11 | +/- |
| Panobinostat | All HDACs | +/- |
| ITF2357 | HDAC Class I and II | + |
| Scriptaid | HDAC 1,3,8 | +++ |
| Nexturastat A | HDAC 6 | +/- |
| PCI-34051 | HDAC 8 | - |
| RGFP966 | HDAC 3 | +/- |
| Pyroxamide | HDAC 1 | +/- |
| Tasquinimod | HDAC 4 | - |
